# Distinct Cytokine Profiles in Severe COVID-19 and Non-Alcoholic Fatty Liver Disease

**DOI:** 10.3390/life12060795

**Published:** 2022-05-26

**Authors:** Neven Papic, Lara Samadan, Nina Vrsaljko, Leona Radmanic, Karlo Jelicic, Petra Simicic, Petra Svoboda, Snjezana Zidovec Lepej, Adriana Vince

**Affiliations:** 1 School of Medicine, University of Zagreb, 10000 Zagreb, Croatia; lsamadan@mef.hr (L.S.); avince@bfm.hr (A.V.); 2 Department for Viral Hepatitis, University Hospital for Infectious Diseases, 10000 Zagreb, Croatia; nvrsaljko@bfm.hr (N.V.); jelicic.karlo@icloud.com (K.J.); 3 Department for Clinical Immunology and Molecular Diagnostics, University Hospital for Infectious Diseases, 10000 Zagreb, Croatia; leona.radmanic@gmail.com (L.R.); petrasimicic@gmail.com (P.S.); szidovec@gmail.com (S.Z.L.); 4 Research Department, University Hospital for Infectious Diseases, 10000 Zagreb, Croatia; psvoboda@bfm.hr; 5 Department of Biology, Faculty of Science, University of Zagreb, 10000 Zagreb, Croatia

**Keywords:** COVID-19, NAFLD, SARS-CoV2, inflammation, cytokines, interleukin-6, interleukin-8, interferons, MAFLD, obesity

## Abstract

Non-alcoholic fatty liver disease (NAFLD) is identified as a risk factor for developing severe COVID-19. While NAFLD is associated with chronic low-grade inflammation, mechanisms leading to immune system hyperactivation remain unclear. The aim of this prospective observational study is to analyze cytokine profiles in patients with severe COVID-19 and NAFLD. A total of 94 patients with severe COVID-19 were included. Upon admission, clinical and laboratory data were collected, a liver ultrasound was performed to determine the presence of steatosis, and subsequently, 51 were diagnosed with NAFLD according to the current guidelines. There were no differences in age, sex, comorbidities, and baseline disease severity between the groups. Serum cytokine concentrations were analyzed using a multiplex bead-based assay by flow cytometry. Upon admission, the NAFLD group had higher C-reactive protein, procalcitonin, alanine aminotransferase, lactate dehydrogenase, and fibrinogen. Interleukins-6, -8, and -10 and CXCL10 were significantly higher, while IFN-γ was lower in NAFLD patients. Patients with NAFLD who progressed to critical illness had higher concentrations of IL-6, -8, -10, and IFN-β, and IL-8 and IL-10 appear to be effective prognostic biomarkers associated with time to recovery. In conclusion, NAFLD is associated with distinct cytokine profiles in COVID-19, possibly associated with disease severity and adverse outcomes.

## 1. Introduction

Since the beginning of the COVID-19 pandemic, a substantial effort has been directed towards identifying high-risk groups for developing severe disease, as well as understanding the immune response and its impact on clinical outcomes. The host immune response to SARS-CoV2 is exceptionally complex and heterogeneous in infected patients [1,2]. If a hyperactivation of the immune response occurs, antiviral interferon responses are blunted with an excessive release of pro-inflammatory cytokines, known as the “cytokine storm”, leading to increased immune cell recruitment and tissue damage all contributing to the development of severe disease [1,2].

There is growing evidence that patients with liver diseases are at increased risk for SARS-CoV2 infection [3]. Non-alcoholic fatty liver disease (NAFLD) is the most common cause of chronic liver disease in the Western population, with a prevalence of about 25–30% [4,5,6]. Patients with NAFLD are at increased risk for SARS-CoV2 infection and hospitalization, independently of other components of metabolic syndrome [5,7,8,9]. Furthermore, NAFLD is associated with increased disease severity, longer hospitalization, and adverse outcomes, including pulmonary thrombosis [8,10]. However, the immunological mechanism by which NAFLD aggravates COVID-19 remains unclear. It has been suggested that NAFLD exacerbates the “cytokine storm” through the hepatic release of pro-inflammatory cytokines [11].

There is growing evidence that NAFLD is a multisystem disease associated with chronic low-grade inflammation, impaired immune responses, and microvascular endothelial dysfunction [12,13]. Several studies showed that patients with NAFLD have intrinsically higher C-reactive protein (CRP) and IL-6 serum concentrations, which are both associated with COVID-19 severity [13,14,15]. However, there have been no data on inflammatory responses in a subgroup of patients with NAFLD, while patients with chronic liver diseases are usually excluded from randomized clinical trials. Therefore, there is a significant gap in our understanding of COVID-19 pathogenesis in patients with NAFLD.

The aim of this prospective observational study is to analyze cytokine profiles in patients with severe COVID-19 and NAFLD to better understand the underlying mechanisms making them predisposed to severe COVID-19.

## 2. Materials and Methods

### 2.1. Study Design and Population

This study is a part of an ongoing prospective observational study that is being conducted at the University Hospital for Infectious Diseases Zagreb (UHID), Croatia (COVID-FAT, ClinicalTrials.gov Identifier: NCT04982328). Ninety-four adult patients hospitalized with confirmed COVID-19 between September and December 2021 were included. At that time, the Delta (B.1.617.2 and AY lineages) SARS-CoV-2 variant predominated in Croatia (data were taken from the ECDC database on SARS-CoV-2 variants [16]). The delta SARS-CoV-2 variant was shown to cause more severe disease [17]. These patients have not been reported in previous studies. The patients were required to have the severe disease at hospital admission, as defined by bilateral pulmonary infiltrates on chest imaging, SpO2 ≤ 94% on room air, and/or dyspnea or respiratory frequency ≥ 24 breaths/min). Patients who developed a critical illness or required ICU admission within the first 48 h were excluded, as well as those who started corticosteroid or antiviral treatment before enrolment. Additional exclusion criteria were history of chronic liver disease, significant alcohol consumption, active malignant disease, pregnancy, and immunosuppression. All participants gave written informed consent. The study conformed to the ethical guidelines of the Declaration of Helsinki and was approved by the UHID Ethics Committee (code 01-673-4-2021).

### 2.2. Laboratory and Clinical Data

Demographic and comorbidity data (including the presence of components of metabolic syndrome, cardiovascular, kidney, and neurological conditions), chronic medications, symptoms, and baseline clinical status were collected at admission. Anthropometric measurements, including body mass index (BMI), waist circumference (WC), waist–hip ratio (WHR), and waist–height ratio (WHtR), were measured in all patients. A BMI over 30 was considered obese. All patients underwent abdominal ultrasound as the principal method to identify and grade liver steatosis. The following blood laboratory data from the routine workup at the admission were collected: C-reactive protein (CRP), procalcitonin (PCT), ferritin, white blood cell count (WBC), absolute neutrophil and lymphocyte count (ANC and ALC, respectively), platelet count (Plt), bilirubin, aspartate aminotransferase (AST), alanine aminotransferase (ALT), blood urea nitrogen (BUN), serum creatinine, gamma-glutamyl transferase (GGT), lactate dehydrogenase (LDH), fibrinogen, and D-dimer levels. Patients were treated according to the standard of care (including remdesivir, dexamethasone, low-molecular-weight heparin, and tocilizumab) and at the discretion of the managing physician. Clinical evolution, including oxygen requirements, invasive and non-invasive ventilation, and complication rates, were assessed daily and collected in a standardized form.

### 2.3. NAFLD Definition

Upon admission, the liver steatosis was assessed by ultrasound in all patients by an experienced radiologist and defined as increased echogenicity and sound attenuation of liver parenchyma [4,6,18]. Patients were subsequently diagnosed with NAFLD according to current guidelines that require: (1) evidence of liver steatosis, (2) no significant alcohol consumption, (3) no competing causes of liver steatosis, and (4) no coexisting causes of chronic liver disease (including viral hepatitis, which was excluded by testing for HCV antibodies and HBsAg) [4,6].

### 2.4. Cytokine Measurement

Serum cytokine responses in 94 patients were analyzed using a multiplex bead-based assay LEGENDplex Human Anti-Virus Response Panel (BioLegend, San Diego, CA, USA), which allows the simultaneous quantification of 13 human biological response modifiers by flow cytometry (FACS Canto II, Becton Dickinson, Franklin Lakes, NJ, USA). The panel included: IFN-α2 and IFN-β (type I interferons), IFN-γ (type II interferon), IFN-λ1 and IFN-λ2/3 (type III interferons), cytokines associated with innate and early pro-inflammatory immune responses (TNF-α, IL-6), a principal inflammatory mediator IL-1β, pro-inflammatory chemokines IL-8 (CXCL8) and IP-10 (CXCL10), immunoregulatory cytokine IL-12p70 important in the differentiation of Th1 type cells, a key anti-inflammatory cytokine IL-10 and multifunctional cytokine GM-CSF. The assays’ minimum detectable concentrations (MDC) in the serum were as follows: IL-1β (1.4 pg/mL), IL-6 (1.0 pg/mL), IL-8 (1.4 pg/mL), IL-10 (0.9 pg/mL), IL-12p70 (1.1 pg/mL), IFN-α (1.3 pg/mL), IFN-β (1.5 pg/mL), IFN-λ1 (IL-29) (1.9 pg/mL), IFN-λ2/3 (IL-28A/28B) (12.8 pg/mL), IFN-γ (0.7 pg/mL), TNF-α (1,0 pg/mL), IP-10 (CXCL10) (2.0 pg/mL), and GM-CSF (1.1 pg/mL). The samples were diluted 2-fold with an Assay Buffer before being tested (as recommended by the manufacturer).

### 2.5. Statistical Analysis

The clinical characteristics, laboratory, and demographic data were evaluated and descriptively presented as frequencies and medians with interquartile ranges. Fisher’s exact test and the Mann–Whitney U test were used to compare the groups. All tests were two-tailed; a *p*-value < 0.05 was considered statistically significant. Correlations were analyzed using Spearman’s rank correlation coefficient and summarized in a correlation matrix. The discriminatory performances of the laboratory variables considered were compared using a receiver operating characteristic (ROC) analysis. Time to hospital discharge or readiness for discharge stratified by cytokine levels was evaluated using the Kaplan–Meier method and hazard ratios (HR) with 95% confidence intervals (95% CI) and *p*-values were calculated by the log-rank test. Statistical analyses were performed using GraphPad Prism Software version 9.3.1 (San Diego, CA, USA).

## 3. Results

### 3.1. Baseline Patients’ Characteristics

A cohort of 94 adult hospitalized patients (55 males, median age of 66, IQR 61–69 years) were included in the study. Of them, 51 were diagnosed with NAFLD. There were no differences in age, sex, and comorbidities between the groups, except for higher BMI and waist–hip ratio in patients with NAFLD, as presented in Table 1. The median time interval from the onset of disease to admission was similar between groups (9, IQR 7–11 days vs. 8, IQR 7–11 days, *p* = 0.4738). Due to the inclusion criteria, all patients on admission were required to have severe, but not critical, COVID-19. Upon admission, the required oxygen supplementation to maintain SpO2 ≥ 90% was similar, with a median of 7 L of O2/min (IQR 3–25).

As shown in Table 2, patients with NAFLD had higher C-reactive protein (119 mg/L, IQR 82–188 vs. 98 mg/L, IQR 38–134), procalcitonin (0.2 µg/L, IQR 0.09–0.42 vs. 0.09 µg/L, IQR 0.07–0.18), alanine aminotransferase (51 IU/L, IQR 34–83 vs. 34 IU/L, IQR 23–57), lactate dehydrogenase (421 IU/L, IQR 320–559 vs. 311 IU/L, IQR 237–475), and fibrinogen (6.4 g/L, IQR 5.6–7.8 vs. 5.8 g/L, IQR 5.3–6.6) on admission. There were no differences in other routine laboratory parameters, except for platelets, which were lower in the NAFLD group (159 × 10^9^/L, IQR 122–226 vs. 217 × 10^9^/L, IQR 151–279).

Patients were treated according to the current standard of care: remdesivir (37, 39.36%), corticosteroids (dexamethasone (88, 93.62%) and/or methylprednisolone (6, 6.38%), LMWH (94, 100%), and tocilizumab (5, 5.32%). There were no differences in the choice of treatment between the groups. The median duration of hospitalization was similar between the groups (10, IQR 7–15 vs. 10, IQR 5–16 days). Regarding the clinical outcomes, 26 (27.66%) patients progressed to critical illness and required intensive-care unit treatment (15, 29.41% in NAFLD and 11, 25.58% in the non-NAFLD group). Twelve patients died (12.76%), five with NAFLD and seven without NAFLD.

### 3.2. Cytokine and Chemokine Concentrations in Patients with and without NAFLD

**Figure 1:**
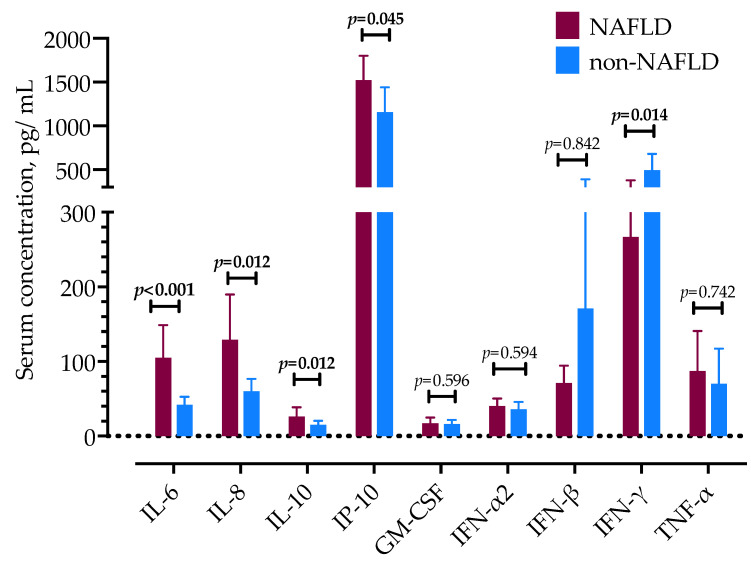
Serum concentrations of selected cytokines in patients with COVID-19, with or without NAFLD. Data are presented as mean with 95% confidence intervals and analyzed by the Mann–Whitney U test.

Next, we examined a panel of cytokines and chemokines associated with anti-viral response in patients with and without NAFLD. Interleukins-6 and -10 were significantly higher in patients with NAFLD, as well as chemokines IL-8 (CXCL8) and IP-10 (CXCL10), as shown in Table 3 and Figure 1. Serum concentrations of IFN-γ were significantly lower in patients with NAFLD. There were no differences in IFN-α2, IFN-β, GM-CSF, and TNF-α levels. Of the other tested cytokines, IL-1β serum concentrations were lower than the detection thresholds in most patients; 12 patients with NAFLD had elevated IL-1β, a median of 82 pg/mL (IQR 72–178), and 7 without NAFLD (85, IQR 34–96 pg/mL). Similarly, IL-12p70 was elevated in 25 patients, more frequently in patients without NAFLD (16 patients, median of 4.0 pg/mL, IQR 3.5–7.9) than in patients with NAFLD (9 patients, 3.5 pg/mL, IQR 2.7–4.9). IFN-λ1 and IFN-λ2/3 were detected in only 2 and 11 patients, respectively.

**Figure 2:**
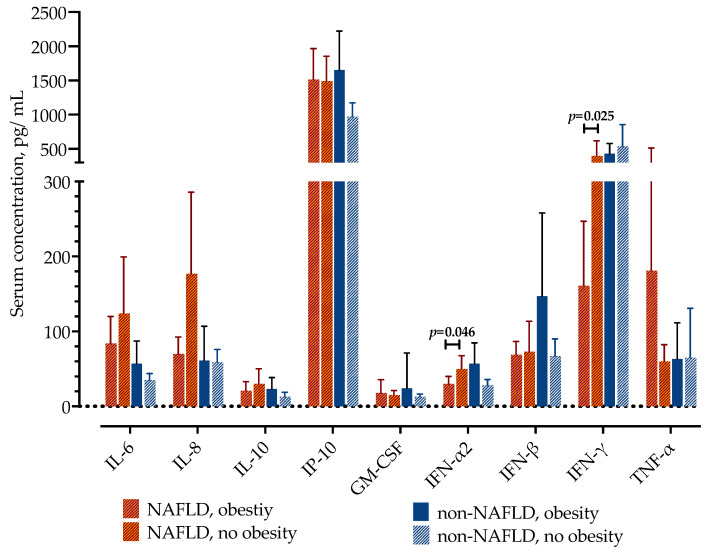
Serum concentrations of selected cytokines in patients with COVID-19 and NAFLD, with or without obesity. Data are presented as mean with 95% confidence intervals and the significance between the two groups was analyzed by the Mann–Whitney U test.

Next, we examined if the differences in cytokine concentrations were caused by the presence of obesity. Except for the lower concentrations of IFN-α2 (22 pg/mL, IQR 14–37 vs. 41 pg/mL, IQR 25–62, *p* = 0.046) and IFN-γ (124 pg/mL, IQR 52–274 vs. 354 pg/mL, IQR 199–597, *p* = 0.025) in patients with NAFLD and obesity, there were no differences in other cytokine concentrations. Meanwhile, in patients without NAFLD, there were no differences in measured cytokine concentrations depending on the presence of obesity, as presented in Figure 2.

### 3.3. Association of Cytokines with COVID-19 Severity in Patients with NAFLD

We further assessed the association of serum cytokine concentrations upon admission with subsequent disease progression during hospitalization. A cohort of patients with NAFLD was divided into two groups: patients who developed critical illness (requirement of NIV/IMV or ECMO) and those with stable disease. Of 51 patients with NAFLD, 15 developed critical illness (29.41%) at a median of 4 days after hospital admission. First, we examined the differences in routine laboratory findings and found that only CRP and LDH were significantly higher in patients who developed the critical disease, as shown in Table 4.

**Figure 3:**
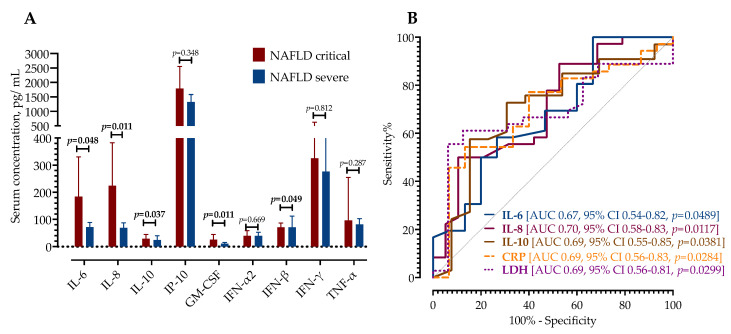
Panel (**A**)—Serum concentrations of cytokines with NAFLD and severe and critical illness. Data are presented as mean with 95% confidence intervals and analyzed by Mann–Whitney U test. Panel (**B**)—ROC curve analysis of IL-6, IL-8, IL-10, CRP, and LDH with corresponding AUC.

Next, we examined the differences in cytokine expression regarding disease severity (Table 4). Patients with critical disease had higher concentrations of IL-6 (94 pg/mL, IQR 53–216 vs. 54, IQR 36–116), IL-8 (94 pg/mL, IQR 53–213 vs. 51, IQR 36–88), and IL-10 (20 pg/mL, IQR 13–38 vs. 12, IQR 6.7–20) upon admission. Similarly, GM-CSF (23 pg/mL, IQR 14–35 vs. 9.6, IQR 7.8–11) and IFN-β (65 pg/mL, IQR 57–80 vs. 56, IQR 42–64) were higher in patients who developed critical COVID-19, as presented in Figure 3, Panel A. There were no differences in other measured cytokines. Meanwhile, patients without NAFLD and critical COVID-19 had higher serum concentrations of CRP (127 mg/L, IQR 75–202 vs. 89 mg/L, IQR 32–122; *p* = 0.016), procalcitonin (0.17 µg/L, IQR 0.11–0.28 vs. 0.09, IQR 0.06–0.12; *p* = 0.006), and LDH (511 IU/L, IQR 419–620 vs. 284, IQR 236–445; *p* = 0.012). However, in the same subgroup of patients, only IFN-α2 (55 pg/mL, IQR 41–74 vs. 25, IQR 14–34; *p* = 0.0216) was significantly higher, while there were no significant differences in serum concentrations of other cytokines.

A receiver operating characteristic (ROC) curve analysis was performed to calculate the area under the curve (AUC) of selected cytokines as diagnostic biomarkers for distinguishing critical from a severe illness in patients with NAFLD. As shown in Figure 3, Panel B, IL-8, IL-10, and IL-6 had an AUC of 0.70, 0.69, and 0.67, respectively.

**Figure 4:**
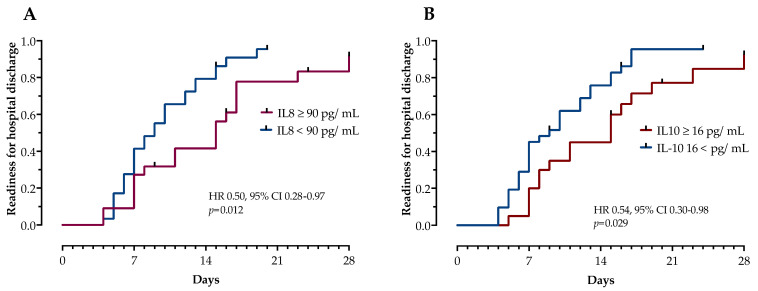
Association of time to recovery with IL-8 (panel (**A**)) and IL-10 (panel (**B**)). Kaplan–Meier curves on “time to recovery” in patients with NAFLD and COVID-19 are stratified by IL-6 and IL-8. Hazard ratios with 95% confidence intervals and *p*-values were calculated by the log-rank test.

Next, we examined the impact of cytokines associated with disease severity in ROC analysis on time to recovery, defined by time to hospital discharge or readiness for discharge. In a survival analysis using Kaplan–Mayer estimates, IL-8 (≥90 pg/mL, log-rank test *p* = 0.012) and IL-10 (≥16 pg/mL, log-rank test *p* = 0.029) appear to be efficient prognostic biomarkers associated with longer time to recovery, as shown in Figure 4. IL-6, CRP, and LDH were not associated with time to recovery in patients with NAFLD.

### 3.4. Correlation Analysis of Serum Biomarkers in Patients with NAFLD

**Figure 5:**
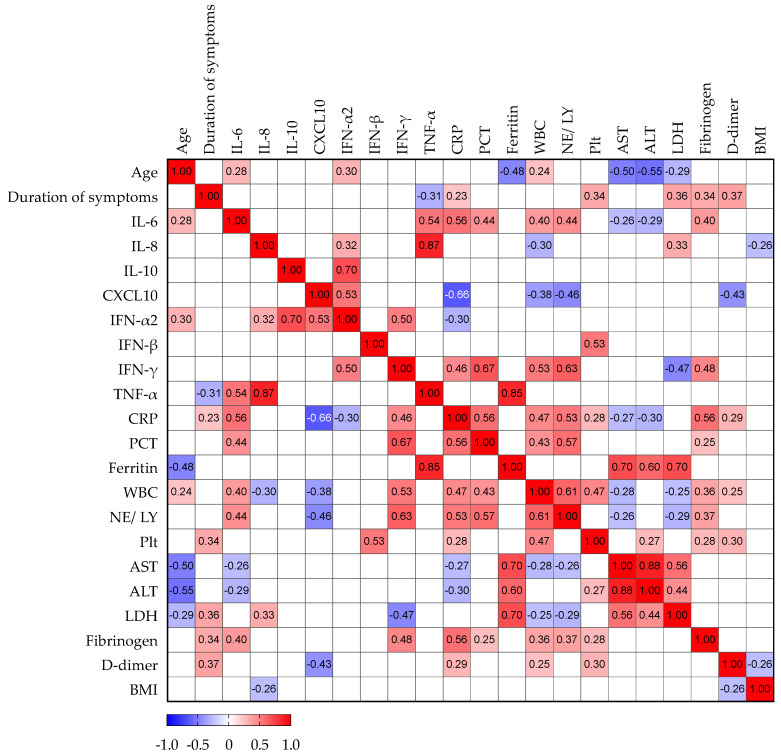
Spearman’s correlation correlogram. The strength of the correlation between two variables is represented by the color at the intersection of those variables. Colors range from dark blue (strong negative correlation; *r* = −1.0) to red (strong positive correlation; *r* = 1.0). Results were not represented if *p* > 0.05.

Next, we analyzed potential correlations among paired laboratory parameters, including cytokine concentrations and clinical variables in patients with NAFLD, as presented in Figure 5. Interestingly, the duration of illness correlated positively with CRP, platelet count, LDH, fibrinogen, and D-dimers, but not with cytokine concentrations. As expected, WBC and CRP positively correlated with IL-6, IFN-γ, PCT, NE/LY ratio, and fibrinogen. WBC showed a negative correlation with IL-8 and CXCL10 and CRP with CXCL10 and IFN-α2. IL-8 is positively correlated with TNF-α and IFN-α2, and negatively with WBC and BMI. IL-10 correlated only with IFN-α2. Aminotransferases, AST, and ALT showed a negative correlation with age, IL-6, CRP, and WBC, and a positive correlation with ferritin and LDH.

## 4. Discussion

In this paper, we provide the first evidence that COVID-19 patients with NAFLD have distinct serum cytokine profiles than patients without NAFLD. This includes higher levels of IL-6, IL-8, IL-10, and CXCL10, and lower IFN-γ. Furthermore, patients with NAFLD who subsequently progressed to critical disease had higher concentrations of IL-6, -8, -10, and IFN-β upon admission, and IL-8 and IL-10 appear to be efficient prognostic biomarkers associated with time to recovery.

In our study, the concentration of IL-6 could differentiate COVID-19 NAFLD from non-NAFLD patients, as well as NAFLD critical from NAFLD non-critical patients. IL-6 has already been described as an independent prognostic factor of COVID-19 severity and mortality [19]. Gao et al., in a case–control study that included 46 Chinese patients with NAFLD and 121 controls from early 2020, showed that patients with NAFLD had higher serum IL-6 levels that correlated with disease severity [15]. As a pleiotropic cytokine, IL-6 plays an important role as an inducer of hepatic acute phase responses, and one of the cytokine-targeted approaches for severe COVID-19 includes IL-6 blockade, however with modest clinical benefit. Since serum CRP and IL-6 are natively elevated in NAFLD patients [20], increased levels in COVID-19 are not an unexpected finding. NAFLD is closely associated with metabolic syndrome and immunologically activated adipose tissue, a major site of IL-6 production [21]. While the possible explanation includes a higher prevalence of obesity in patients with NAFLD, our results showed no differences in IL-6 concentrations between non-obese and obese NAFLD patients. Furthermore, we found no correlations between IL-6 levels and WHR or WhTR (data not shown), suggesting other possible immunological mechanisms.

Next, we found significantly higher IL-8 concentrations in patients with NAFLD that were associated with time to recovery. Interestingly, according to some studies, IL-8 showed a better correlation than serum IL-6 levels in predicting COVID-19 severity and mortality [22,23]. There are some pathological similarities between the progression of NAFLD and the development of acute respiratory distress syndrome (ARDS) in COVID-19. Briefly, the infiltration of the liver by activated neutrophils is one of the key events that contribute to disease progression from NAFLD to non-alcoholic steatohepatitis (NASH) [24]. Just like a neutrophil-to-lymphocyte ratio in the peripheral blood is a prognostic factor for disease severity and mortality in COVID-19, it is a useful prognostic biomarker marker for the development of NASH and fibrosis in NAFLD [25,26]. Activated neutrophils synthesize IL-8, a chemotactic cytokine that recruits and activates additional neutrophils, but also recruits T cells and basophils to sites of inflammation [24]. Indeed, circulating IL-8 levels were associated with NASH and the progression of fibrosis [27]. Therefore, it is likely that the increased concentrations of IL-8 in the NAFLD group are closely associated with the contribution of activated neutrophils to the pathogenesis of the disease.

Interestingly, we found increased concentrations of CXCL10 (or IP-10) and decreased IFN-γ in patients with NAFLD. CXCL10 is a primary IFN-γ-inducible immunomodulatory cytokine with a role in homing activated Th1 CD4+ T cells to sites of infection or inflammation [28]. The synthesis of IFN-γ by Th1 CD4+ T cells induces the de novo synthesis of CXCL10 and perpetuates the inflammatory immune responses [29]. Meanwhile, IFN-γ is a Th1-type cytokine that exhibits antiviral, antiproliferative, and immunomodulatory activity [30]. Low IFN-γ concentrations are a poor prognostic factor in COVID-19 and are associated with an increased chance of developing pulmonary fibrosis [30,31]. Contrarily, elevated levels of CXCL10 were reported to be associated with COVID-19 severity and mortality [32]. This suggests that patients with NAFLD and COVID-19 might have different regulations of CXCL10 secretion. The decreased IFN-γ concentrations are most likely associated with the functional impairment of NK-cells (main cellular sources of IFN-γ) that were previously described in NAFLD [33]. Recent gene expression analysis identified CXCL10 as one of the five candidate therapeutic targets for the treatment of NAFLD, highlighting its role in NAFLD progression [34]. CXCL10 might represent a possible target for innovative treatment strategies of COVID-19 as well.

The increased concentrations of IL-10 observed in our research are in concordance with recent observations on immune dysregulation in NAFLD that includes modulation of inflammatory and adipocytokine levels and altered Th17/Treg balance [35]. Furthermore, studies have shown that proinflammatory cytokines (such as IL-6, IL-12, and TNF- α) are associated with the insufficient modulation of IL-10 [36]. In severe COVID-19, an early increase in IL-10 concentration was observed [37]. This could be explained as a failed attempt to suppress the hyperinflammatory response and tissue damage since IL-10 concentrations strongly correlated with IL-6 and other inflammatory markers, such as CRP [37]. There is also a possibility of “IL-10 resistance” in which activated immune cells escape the anti-inflammatory IL-10 signaling, thereby enhancing the hyperinflammatory response [38,39].

Our study should be viewed within its limitations: the diagnosis of NAFLD was based on an abdominal ultrasound, which is operator-dependent; some of the cytokines measured were below the detection range probably due to the test performances and their impact could not be analyzed (IFNs lambda, IL-1β, and IL-12p70); the cytokine concentrations were examined at a single timepoint and dynamic changes in cytokine responses were not analyzed; and patients with less severe COVID-10 were not included. Nevertheless, we studied a relatively large and well-defined cohort of patients and report the first data examining the cytokine profile in patients with NAFLD and severe COVID-19. COVID-19 is an immunologically “demanding” disease, involving all components of the immune system, creating dysregulation of immune response with cytokine storms and the simultaneous detection of pro- and anti-inflammatory cytokines and chemokines. In COVID-19 patients with NAFLD, the situation is even more complicated due to liver involvement. Surprisingly, while the clinical association of NAFLD with COVID-19 severity is well described and the possible underlying immunological mechanisms have been reviewed, there are no studies examining the proposed immunological hypothesis.

## 5. Conclusions

In conclusion, we showed that patients with NAFLD have a different immune response to severe COVID-19, with IL-6, IL-8, IL-10, and CXCL10 as a possible culprits of an uncontrolled inflammation associated with disease severity in this group of patients. Identifying the distinct cytokine profile in patients with COVID-19 and NAFLD could have practical prognostic implications and could initiate new therapeutic strategies based on a better understanding of the patient’s immune response.

## Figures and Tables

**Table 1:** Baseline patients’ characteristics.

	NAFLD (*n* = 51)	Non-NAFLD (*n* = 43)	Difference (95% CI) ^c^	*p*-Value ^a^
Age, median (IQR ^b^)	62 (51–67)	60 (45–66)	−2 (−7 to 3)	0.488
Male, No. (%)	33 (64.71%)	22 (51.16%)	13.5% (−7.6 to 33.4%)	0.219
Overweight (BMI 25–30 kg/m^2^)	22 (43.14%)	11 (25.58%)	17.6% (−3.3 to 36.1%)	0.087
Obesity (BMI > 30 kg/m^2^)	24 (47.06%)	12 (27.90%)	19.15% (0.4 to 40.3%)	0.088
BMI (kg/m^2^)	30 (27–34)	27 (25–30)	−2.7 (−4.8 to −0.77)	**0.005**
Waist–hip ratio	1 (1–1.1)	0.97 (0.92–1)	−0.05 (−0.09 to 0.003)	**0.035**
Diabetes Mellitus	16 (31.37%)	8 (18.60%)	12.7% (−6.5 to 30.3)	0.235
Arterial Hypertension	29 (56.86%)	17 (39.53%)	17.3% (−4.3 to 36.9%)	0.103
Gastritis/GERD	2 (3.92%)	2 (4.65%)	1.6% (−10.3 to 12.5%)	>0.999
Dyslipidemia	10 (19.61%)	5 (11.63%)	7.9% (−9.0 to 23.7%)	0.399
Cardiovascular Disease	4 (7.84%)	3 (6.98%)	0.8% (−13.3 to 13.8%)	>0.999
Duration of illness, days	9 (7–11)	8 (7–11)	0 (−2 to 1)	0.473
Body temperature, °C	37 (37–38)	38 (37–39)	0.4 (−0.1 to 0.9)	0.086
Dyspnea	28 (54.90%)	21 (48.84%)	6.1 (−15.1 to 26.6)	0.679
Respiratory rate, /min	24 (20–29)	28 (23–30)	2.0 (0 to 4.0)	0.180
Heart rate, /min	90 (85–103)	97 (84–109)	4.0 (−5.0 to 10)	0.396
Oxygen saturation (SpO2) on room air, %	89 (87–91)	91 (87–92)	1.0 (0 to 3)	0.099

^a^ Fisher’s exact or Mann–Whitney U test, as appropriate; ^b^ IQR, interquartile range; ^c^ presented are standardized differences among medians or proportions with corresponding 95% confidence intervals (95% CI).

**Table 2:** Laboratory findings on admission.

	NAFLD (*n* = 51)	Non-NAFLD (*n* = 43)	Difference (95% CI) ^c^	*p*-Value ^a^
CRP, mg/L, median (IQR ^b^)	119 (82–188)	98 (38–134)	−39 (−66 to 6.4)	**0.019**
Procalcitonin, µg/L	0.2 (0.09–0.42)	0.09 (0.07–0.18)	−0.07 (−0.14 to −0.025)	**0.001**
Ferritin, µg/L	688 (437–1676)	907 (552–1399)	70 (−407 to 425)	0.798
WBC, ×10^9^/L	6.0 (5.0 to 10.0)	6.6 (5.0 to 8.4)	−0.1 (−1.5 to 1.1)	0.881
Lymphocyte count, 10^9^/L	0.74 (0.58–1.1)	0.68 (0.5–1.0)	−0.06 (−0.20 to 0.11)	0.475
Neutrophils/ lymphocytes ratio	6.5 (4.4–12)	6.6 (4.5–11)	0.21 (−1.8 to 2.1)	0.844
Hemoglobin, g/L	137 (131–147)	137 (125–147)	−4 (−10 to 2.0)	0.227
Platelets, ×10^9^/L	159 (122–226)	217 (151–279)	42 (8.0 to 77)	**0.018**
Bilirubin, µmol/L	12 (10–16)	10 (9–14)	−1 (−3.0 to 0)	0.093
AST, IU/L	53 (38–79)	51 (31–83)	−4 (−17 to 9)	0.512
ALT, IU/L	51 (34–83)	34 (23–57)	−13 (−24 to −3)	**0.013**
GGT, IU/L	52 (26–102)	44 (30–70)	−3 (−19 to 10)	0.709
LDH, IU/L	421 (320–559)	311 (237–475)	−83 (−155 to −10)	**0.029**
Fibrinogen, g/L	6.4 (5.6–7.8)	5.8 (5.3–6.6)	−0.6 (−1.3 to 0.10)	**0.023**
D-dimer, mg/L	1.2 (0.71–2.1)	0.88 (0.61–1.7)	−0.15 (−0.48 to 0.13)	0.278

^a^ Fisher exact or Mann–Whitney U test, as appropriate; ^b^ IQR, interquartile range; ^c^ presented are standardized differences between medians or proportions with corresponding 95% confidence intervals (95% CI). Abbreviations: C-reactive protein (CRP), white blood cell count (WBC), aspartate aminotransferase (AST), alanine aminotransferase (ALT), gamma-glutamyl transferase (GGT), lactate dehydrogenase (LDH).

**Table 3:** Serum concentrations of selected cytokines in patients with and without NAFLD.

	NAFLD (*n* = 51)	Non-NAFLD (*n* = 43)	Difference (95% CI) ^c^	*p*-Value ^a^
Interleukin-6, pg/mL ^b^	67 (40–120)	34 (17–56)	−32 (−54 to −16)	**<0.001**
Interleukin-8, pg/mL	62 (40–116)	49 (27–64)	−20 (−39 to −4.4)	**0.012**
Interleukin-10, pg/mL	13 (9.6–25)	9.3 (3.8–16)	−4.6 (−8.6 to −1.1)	**0.012**
IFN-α2, pg/mL ^d^	33 (20–54)	27 (15–48)	−4.3 (−14 to 6.5)	0.539
IFN-β, pg/mL ^e^	64 (49–72)	56 (49–78)	0 (−14 to 14)	0.842
IFN-γ, pg/mL	260 (57–354)	384 (260–639)	191 (30 to 331)	**0.014**
IP-10 (CXCL10), pg/mL	1355 (694–1990)	932 (595–1509)	−334 (−652 to −4.6)	**0.045**
GM-SCF, pg/mL ^f^	10 (7.8 to 25)	13 (9.6 to 17)	1.1 (−6.1 to 5.5)	0.596
TNF-α, pg/mL ^f^	60 (38–108)	49 (21–129)	−11 (−53 to 54)	0.742

^a^ Fisher’s exact or Mann–Whitney U test, as appropriate; ^b^ IQR, interquartile range; ^c^ presented are standardized differences among medians with corresponding 95% confidence intervals (95% CI). Serum concentrations were within the detection range in ^d^ 60, ^e^ 43, and ^f^ 29 patients.

**Table 4:** Laboratory parameters and serum concentrations of selected cytokines in patients with NAFLD and critical or non-critical illness.

	Critical Illness (*n* = 15)	Non-Critical Illness (*n* = 36)	Difference (95% CI) ^c^	*p*-Value ^a^
Age, median (IQR ^b^)	64 (58–67)	61 (48–67)	−2.0 (−9.0 to 3.0)	0.365
Male sex, No. (%)	12 (80%)	21 (58.33%)	21.67% (−10.9 to 44.5%)	0.202
BMI (kg/m^2^)	30 (28–34)	30 (27–34)	0.47 (−2.1 to 3.2)	0.752
Duration of illness on admission, days	9.5 (8.0–12)	9 (7–11)	−1.0 (−3.0 to 1.0)	0.329
CRP, mg/L	172 (107–205)	99 (71–161)	−46 (−95 to 6.6)	**0.023**
Procalcitonin, µg/L	0.27 (0.20–0.57)	0.14 (0.097–0.39)	−0.11 (−0.22 to 0.018)	0.101
Ferritin, µg/L	1051 (456–2345)	684 (456–1772)	−84 (−764 to 327)	0.531
WBC, ×10^9^/L	7.1 (5.2–11)	5.8 (4.5–9.8)	−1.3 (−4.2 to 0.4)	0.102
Lymphocyte count, 10^9^/L	0.74 (0.57–1.1)	0.75 (0.58–1.1)	0.03 (−0.18 to 0.28)	0.739
Neutrophil/lymphocyte ratio	8.4 (6.1–14)	5.6 (3.9–11)	−2.6 (−6.2 to 0.30)	0.069
AST, IU/L	56 (40–140)	49 (32–79)	−11 (−34 to 10)	0.276
ALT, IU/L	40 (27–123)	42 (25–81)	−3.0 (−21 to 15)	0.757
LDH, IU/L	466 (415–660)	364 (289–514)	−103 (−211 to −17)	**0.029**
Fibrinogen, g/L	7.0 (5.5–8.4)	6.2 (5.6–7.8)	−0.30 (−1.3 to 0.6)	0.501
D-dimer, mg/L	1.4 (0.99–3.5)	1.0 (0.70–2.0)	−0.51 (−1.2 to 0.06)	0.095
Interleukin-6	94 (53–216)	54 (36–116)	−38 (−86 to 0)	**0.048**
Interleukin-8	94 (53–213)	51 (36–88)	−36 (−110 to −12)	**0.011**
Interleukin-10	20 (13–38)	12 (6.7–20)	−8.5 (−16 to 0.56)	**0.037**
IFN-α2	37 (20–61)	31 (18–51)	−4.3 (−25 to 16)	0.669
IFN-β	65 (57–80)	56 (42–64)	−14 (−32 to 0)	**0.049**
IFN-γ	249 (137–483)	292 (57–376)	−19 (−296 to 196)	0.813
IP−10 (CXCL10)	1585 (597–2123)	1120 (708–1874)	−266 (−896 to 320)	0.348
GM-SCF	23 (14–35)	9.6 (7.8–11)	−12 (−19 to 1.8)	**0.011**
TNF-α	38 (32–192)	71 (44–116)	22 (−200 to 85)	0.287

^a^ Fisher’s exact or Mann–Whitney U test, as appropriate; ^b^ IQR, interquartile range; ^c^ presented are standardized differences between medians or proportions with corresponding 95% confidence intervals (95% CI).

## Data Availability

The datasets generated during and/or analyzed during the current study are available from the corresponding author upon reasonable request.
